# Design and Evaluation of a Prescription Drug Monitoring Program for Chinese Patent Medicine based on Knowledge Graph

**DOI:** 10.1155/2021/9970063

**Published:** 2021-07-16

**Authors:** Wangping Xiong, Jun Cao, Xian Zhou, Jianqiang Du, Bin Nie, Zhijun Zeng, Tianci Li

**Affiliations:** ^1^School of Computer, Jiangxi University of Chinese Medicine, Nanchang 330004, Jiangxi, China; ^2^Jiangxi Province Key Laboratory of TCM Etiopathogenisis, Jiangxi University of Chinese Medicine, Nanchang 330004, Jiangxi, China

## Abstract

**Background:**

Chinese patent medicines are increasingly used clinically, and the prescription drug monitoring program is an effective tool to promote drug safety and maintain health.

**Methods:**

We constructed a prescription drug monitoring program for Chinese patent medicines based on knowledge graphs. First, we extracted the key information of Chinese patent medicines, diseases, and symptoms from the domain-specific corpus by the information extraction. Second, based on the extracted entities and relationships, a knowledge graph was constructed to form a rule base for the monitoring of data. Then, the named entity recognition model extracted the key information from the electronic medical record to be monitored and matched the knowledge graph to realize the monitoring of the Chinese patent medicines in the prescription.

**Results:**

Named entity recognition based on the pretrained model achieved an F1 value of 83.3% on the Chinese patent medicines dataset. On the basis of entity recognition technology and knowledge graph, we implemented a prescription drug monitoring program for Chinese patent medicines. The accuracy rate of combined medication monitoring of three or more drugs of the program increased from 68% to 86.4%. The accuracy rate of drug control monitoring increased from 70% to 97%. The response time for conflicting prescriptions with two drugs was shortened from 1.3S to 0.8S. The response time for conflicting prescriptions with three or more drugs was shortened from 5.2S to 1.4S.

**Conclusions:**

The program constructed in this study can respond quickly and improve the efficiency of monitoring prescriptions. It is of great significance to ensure the safety of patients' medication.

## 1. Background

Drug safety is an important livelihood issue of concern to countries all over the world. According to the data of WHO [1], one in seven hospitalized patients worldwide each year is due to prescription safety issues. Hospitalizations for drug reactions account for nearly 30% of all hospitalizations in the United States, with approximately 6% of their deaths [2, 3]. At least one out of every eight hospitalized patients in the UK is caused by the wrong medication or problems with the medication itself [4, 5]. Approximately 5% of hospital admissions in developing countries are due to adverse drug reactions, and another 10–20% of hospitalized patients had adverse drug reactions [6, 7]. The phenomenon of irrational drug use in China also cannot be ignored. With the continuous improvement of the level of medicine, the variety and quantity of clinical drugs are increasing, and the probability of drug combination is rapidly increasing. Drug-induced diseases are serious due to the neglect of the rational use and interaction of drugs [8].

Chinese patent medicines (CPM) are widely used because of their stable properties, definite curative effects, relatively small side effects, and convenient administration. However, there are many problems in clinical prescribing [9, 10]. These problems include irregularities in prescribing behavior or repeated use of drugs when patients are treated in different departments and hospitals. When prescribing CPM, doctors ignore the diagnosis and treatment of Chinese medicine. They do not consider the physical characteristics of special populations, including the elderly, children, pregnant, and lactating women, etc., or do not consider the damage to the patients' liver and kidney function caused by the amount of CPM. Doctors ignore the contraindications of CPM and the interaction between medicines.

Resolving the current irrational use of drugs through the development of a safe drug monitoring program has become an effective method for modern pharmaceutical information services [11–13]. This not only helps clinical professionals obtain pharmaceutical information but also simulates the prescription review process and automatically monitors the prescription. This can effectively prevent the occurrence of adverse drug events and promote safe medication use.

## 2. Related Work

Developed countries in Europe and the US first embed prescription drug monitoring programs into electronic prescription systems for real-time regulatory control. European countries have established the European Antimicrobial Resistance Surveillance System (EARSS) and the European Surveillance of Antimicrobial Consumption (ESAC). Boston Hospital introduced the experience and results of clinical pathways into the Prescription Automatic Screening System (PASS). First DataBank, the world's largest drug information database development center, provides comprehensive technical support and data sources for the PASS [14, 15]. The main prescription monitoring systems applied in China are Sichuan Meikang Pharmaceutical's PASS rational drug use monitoring system and Shanghai Datong Pharmaceutical Information Technology Co. This system monitors medication dosage, drug contraindications, interactions, and other factors that may cause physical harm to patients. Real-time monitoring and reminding are performed to avoid medical accidents [16–18].

TCM has been developed for thousands of years, and the knowledge of TCM is constantly emerging. However, it lacks a unified description and completeness of the knowledge system, which makes it difficult to use and share information. In particular, the adverse reactions of CPM have the characteristics of many kinds of drugs, a wide application range, complex components, inconsistent understanding, and nonstandardized naming, etc. [19, 20]. The current basic rule database used in the monitoring framework is mainly for western medicine [21, 22]. There is a lack of standardized data rule bases such as contraindications to the combined use of CPM and evidence-based treatment. Therefore, the establishment of a complex rule base for monitoring the CPM in prescription urgently needs the support of an information method system adapted to its characteristics.

The knowledge graph is a visual representation of the core structure, frontier fields, and overall knowledge structure and is a method system to achieve the goal of multidisciplinary integration [23–25]. It meets the requirements for a unified description of TCM knowledge and multiscale incomplete information integration and can provide technical support for the monitoring of the rational use of CPM. In recent years, scholars have made attempts and explorations in the construction methods and standardization processes of TCM knowledge graph. Yu and Liu [26] proposed the concept of constructing a large-scale knowledge graph based on the TCM Language System (TCMLS) as a framework, and the existing terminology and database resources in the field of TCM as the content and carried out exploration and practices. However, the effective integration of knowledge resources of traditional Chinese medicine has not been realized, and comprehensive, timely, and reliable knowledge services cannot be provided. Tong et al. [27] proposed a semiautomated construction process of knowledge graphs in the field of Chinese medicine knowledge question and answer and auxiliary prescription based on text extraction, relational data conversion, and data fusion technologies.

Zhang et al. [28] proposed an ontology-based representation of the core knowledge graph of Chinese medicine and its construction method. They explored the mapping method between the ontology of Chinese medicine and the knowledge graph and provided a more systematic method and process for the construction of the Chinese medicine knowledge graph. However, the research on the acquisition technology of multisource data and the actual clinical diagnosis and treatment data of traditional Chinese medicine doctors is not in-depth research. Wang et al. [29] took the visualization of chronic gastritis data of traditional Chinese medicine as an example and introduced the random forest technology to visualize the previsual data preprocessing. In general, the knowledge graph theory in TCM is still at the stage of the macro overview on the structure of each discipline. It is urgent to solve the strategy and technology of knowledge graph modeling for the deep integration of multilayer information.

In this paper, we formed a rule base for monitoring CPM by associating disease entities, disease entities, and CPM drug entities through knowledge graphs. The program extracted key information on symptoms, diseases, and medicines in prescriptions through named entity recognition technology and matched them with the existing knowledge base in the knowledge graph. This program can monitor five aspects of prescriptions involving the combined use of CPM, repeated use of medicines, medicines and diseases, the dosage of medicines, and evidence-based treatment.

## 3. Methods

The overall design of this paper is based on a knowledge graph-based rational drug use rule base library and builds a prescription drug monitoring program for CPM. The method is to extract information on CPM, diseases, and conditions through information extraction techniques [30, 31] from the national pharmacopoeia, authoritative data, and high-quality electronic medical record groups. According to the entities and relations of CPM, a knowledge graph was constructed to form a rule base for monitoring the use of CPM.

**Figure 1:**
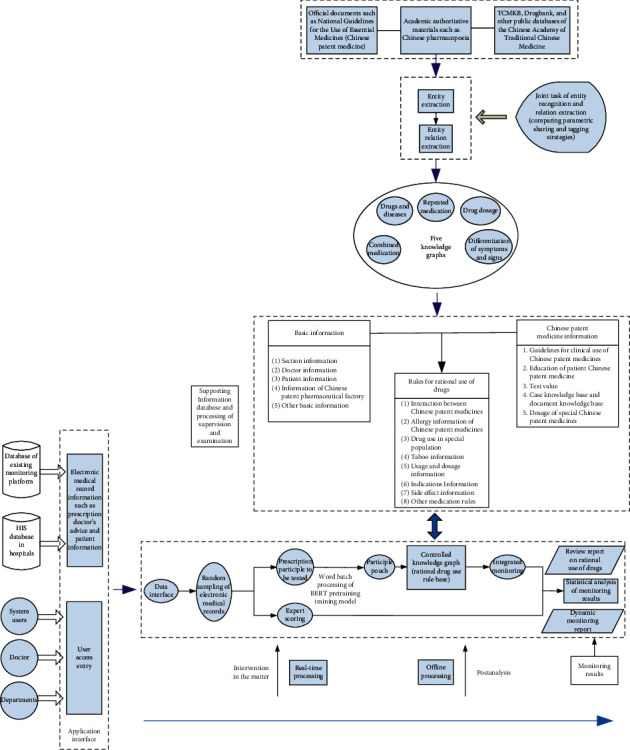
Design of prescription drug monitoring program for CPM.

For the electronic medical records to be monitored, the information related to CPM in the medical records is identified through BERT [32] pretraining model word segmentation [33] and entity recognition. The obtained information is matched with the constructed knowledge graph to monitor the combined use of CPM, repeated use of medicines, medicine and disease, medicine dosage, and evidence-based treatment. The framework design of the prescription drug monitoring program for CPM is shown in Figure 1.

### 3.1. Knowledge Graph

In the knowledge graph, entities are used to represent nodes in the graph, and relations are used to represent edges. In the fields related to Chinese medicine, CPM, diseases, and symptoms can be used as entities, and the relationships between them can connect the corresponding entities to form a relational network library.

The construction and application of a certain scale of knowledge base or rule base require a variety of intelligent information processing technical support. After entity extraction, relationship extraction, knowledge representation, knowledge fusion, etc., the professional domain knowledge graph is formed. In this study, the knowledge base construction work focuses on knowledge extraction, using relational extraction techniques to extract key information of CPM, diseases, and symptoms from the corpus. Finally, it is corrected into a knowledge base and a rule base through manual assistance to serve the prescription drug monitoring program of CPM.

### 3.2. Preprocessing of Medical Records to Be Monitored Based on the BERT Model

This paper mainly analyzed a large number of medical records with CPM prescriptions from local medical institutions in the past five years. There was little medical information in the outpatient medical records and patient's treatment results. Therefore, we mainly monitored the course records and discharge summary in the hospitalized medical records. This was also the main data object for word segmentation of electronic medical records and extraction of medical entities in this paper. In addition, there is no uniform standard for the description of medical records, which is strongly personalized by physicians. Therefore, it is urgent to develop an effective and appropriate method for extracting words and entities.

The BERT model launched by Google in 2018 is a bidirectional encoder representation based on Transformer [32]. When the bidirectional representation model processes a word, it can simultaneously use both the information of the preceding word and the following word. This bidirectional encoding of information makes BERT more suitable than other language models for monitoring a large number of electronic medical records and complex semantics.

Based on the pretraining model, the uploaded electronic medical records are processed for language segmentation and entity extraction. First, based on the maximum probability path of word frequency, the professional dictionary (symptoms, diseases, CPM) was organized to improve the accuracy of word segmentation. Further data screening and entity extraction are required after the word segmentation. We extracted the entities in the electronic medical record and stored them in an array. The values of the array were passed to the constructed rule base module for a matching search of the rule base to achieve the effect of monitoring the CPM in the prescription.

## 4. Results

### 4.1. Knowledge Graph Construction

#### 4.1.1. Knowledge Graph of the Rational Use of CPM

**Figure 2:**
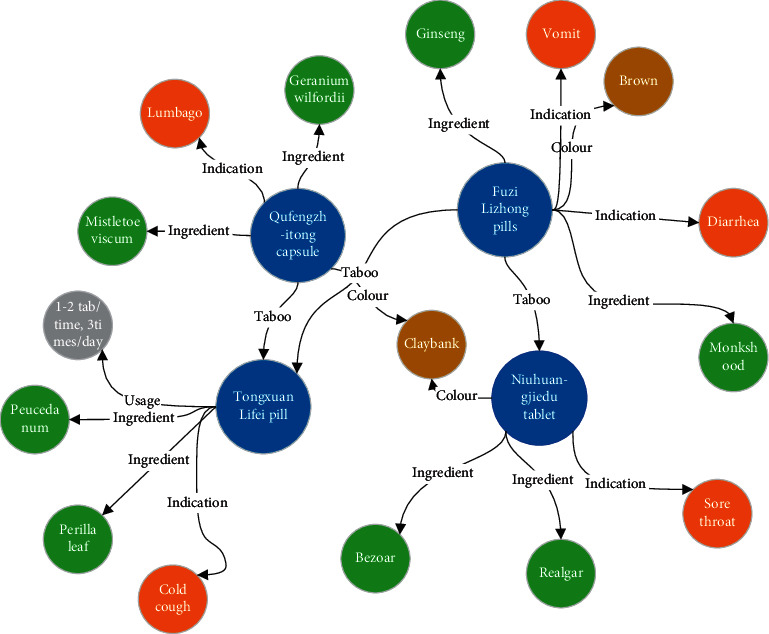
Knowledge graph of rational use of CPM.

The core of rational use of CPM is “eighteen contraindications and nineteen fears.” The Pharmacopoeia of the People's Republic of China clearly indicates that the varieties in the “Eighteen Antibodies” and “Nineteen Fears” are taboos. We have sorted out the contents of the eighteen contraindications and nineteenth fears in the book written by Pang Chunyan. Combining the pharmacopoeia data, it is compiled into a knowledge graph of the rational use of CPM. The drug relationship of some CPM is shown in Figure 2.

#### 4.1.2. Knowledge Graph of Repeated Efficacy Medication

Repeated efficacy medication refers to the simultaneous use of different CPM with the same active ingredients and whether the combination of Chinese and Western medicines is reasonable.

The project designed in this paper mainly adopted the National Essential Medicine (Chinese Medicine) Clinical Guide, Zhang Hongchun's Chinese Medicine Clinical Application Guide-Respiratory Diseases Volume, and the data obtained by crawlers to sort out the results of the knowledge graph.

#### 4.1.3. Knowledge Graph of Disease Symptoms

**Figure 3:**
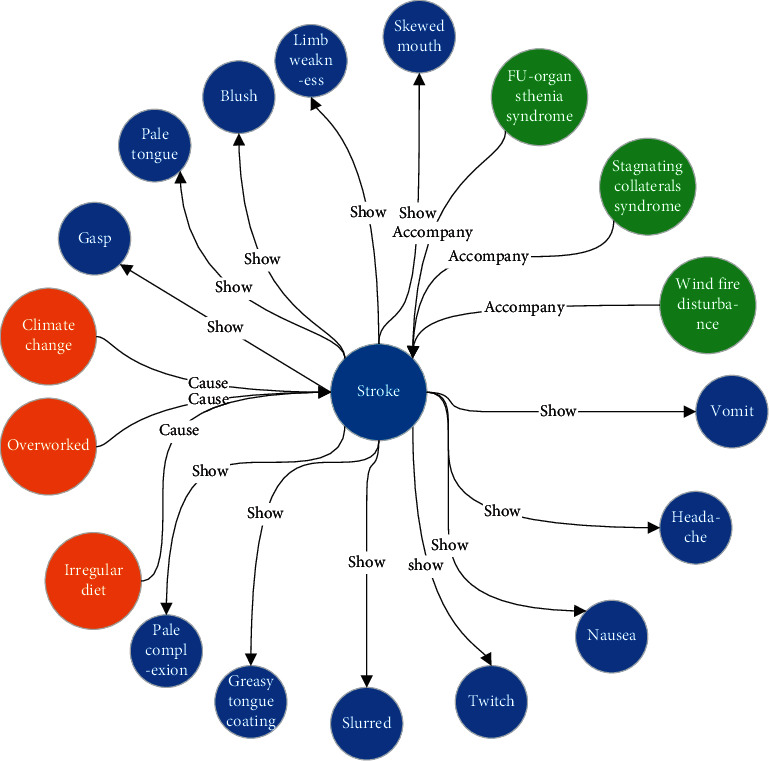
Knowledge graph of disease symptoms.

There are many kinds of Chinese patent medicines and diseases, which cause obstacles for doctors to use medicines. The symptoms corresponding to different diseases are different, and the relationship between the two is many-to-many. We transformed the original unstructured data into structured data and showed the connection between symptoms and diseases by means of knowledge graphs. This can more accurately determine the connection between diseases and symptoms. The knowledge graph shows a wide variety of symptoms, and one disease corresponds to dozens of symptoms.  Figure 3 shows the relationship between stroke, symptoms, and contributing factors in the disease knowledge gap.

### 4.2. Electronic Medical Record Word Segmentation and Entity Recognition

There is more word segmentation in professional fields than in general fields. In order to label the CPM words more accurately, it was necessary to use a self-built dictionary for word segmentation. Then, when labeling the data of CPM, three entity categories were defined.

After defining the entity categories, the National Essential Drug Clinical Application Guide was selected as the original data source. Then, the raw data was turned into word annotated form according to BIO rules for model training. The entity label starts with B, ends with I, and irrelevant text is marked as O. According to the labeling strategy, there were more than 1.7 million characters in total.

We used the BERT pretraining model for named entity recognition training. The evaluation metrics of the model consisted of precision, recall, and F1. The results are shown in Table 1.

For the electronic medical records to be monitored, the model files obtained through training were used to predict the entities in the electronic medical records related to CPM, diseases, and symptoms. These entities were retrieved with the matching of the rule base to achieve the monitoring of CPM in prescriptions.

For the electronic medical records to be monitored, predictions will be made using the model files obtained from the training to obtain the entities in the electronic medical records related to CPM, diseases, and symptoms, which will then be retrieved with the matching of the rule base to achieve the monitoring of CPM in prescriptions.

### 4.3. Framework Applications

The program mainly includes functional modules, such as rational drug knowledge base, rational drug use review, drug dynamic monitoring, and expert prescription review.

**Figure 4:**
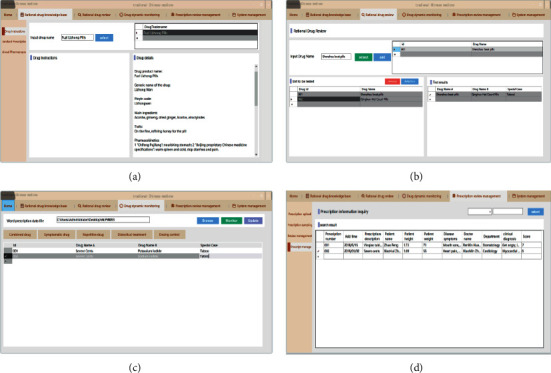
Program applications.

The rational drug knowledge base is to retrieve the drug data in the database and display basic information on the interface. Rational drug dynamic monitoring is introduced to the prescription document to judge whether the prescription is reasonable through monitoring of all aspects. The results are reviewed and confirmed by experts. The program application is shown in Figure 4.

### 4.4. Program Evaluation

To verify the usefulness of the prescription drug monitoring program for CPM, a total of 3,000 electronic medical records involving CPM were monitored and analyzed in 150 sessions in respiratory medicine and pediatrics. Comparing with the indicators of the traditional monitoring program, the indicators of the electronic medical record in this paper were processing response time, offline monitoring of the number of one-time electronic medical records and the accuracy of combined medication monitoring, symptomatic drug administration monitoring, repeat efficacy medication monitoring, evidence-based treatment monitoring, and medication dosage monitoring of CPM. The results are shown in Table 2.

In Table 2, there is no big difference in the accuracy rate of the combined drug monitoring of the two drugs, but the accuracy rate of the combined drug monitoring of three or more drugs is significantly greater than that of the relational database, from 68% to 86.4%. For example, when multidrug combination monitoring is performed in a relational database, multiple tables are associated through fields, which involve the combination of ingredients, toxic ingredients, drug master signs and symptoms contraindications, drug chemical reactions, and other aspects of the synthesis, and then the final monitoring results are obtained. In the association process, the amount of data corresponding to each drug is inconsistent, leading to gaps in the final data missing, which affects the accuracy of monitoring. The graphical database integrates multiscale information together, which can well handle complex and diverse association analysis and meet the analysis and monitoring of the relationship between the combined use of CPM.

Dosage monitoring of CPM should be integrated with diseases and populations, etc. The interactive exploratory analysis based on the knowledge graph can simulate the human thinking process to discover and verify the dosage of CPM in prescriptions. Its accuracy rate has increased from 70% for the traditional program to 97% for the program in this study.

In addition, online prescribing of three or more drugs with conflicting response times of 1.4 seconds in this paper is quicker than that of 5.1 seconds in a relational database. Compared with the traditional storage method, the graphical data storage is quicker in data retrieval and enables real-time decision making in the process of monitoring CPM in prescriptions. Moreover, the query of the relational database is complicated, slow, and beyond expectations, and whose support for joins between nodes is not very friendly. The graphical database represented by the knowledge graph can meet the design requirements based on the characteristics of CPM.

## 5. Conclusions

The prescription drug monitoring program for CPM is based on the basic features and requirements of CPM clinical safety with knowledge graph technology to standardize information on scientific, authoritative, and updated medical and pharmacological knowledge. According to the uploaded electronic medical records, a number of basic reviews of doctors' prescriptions are carried out to realize prescription monitoring and ensure safe medication. The program can effectively regulate the prescribing behavior of physicians and reduce the incidence of irrational use of CPM. This is of great significance to the rational use of drugs in clinical practice and the improvement of medication safety.

In the future work, we will further improve the rational drug use knowledge base and rule base in this paper, including full species drug interaction rule base, drug-food interaction rule base, population contraindication rule base, etc. In addition, an automatic retrieval method for unreasonable drug prescriptions, including prescription medical information, electronic medical records, medical subject thesaurus, and other information, will be established. It can realize active search to locate adverse drug reaction events, and unreasonable drugs use information.

## Figures and Tables

**Table 1:** Metrics of entity recognition.

	DIS (disease)	SYM (dymptom)	ZCY (CPM)	Average
Precision	78.98	82.82	86.57	82.79
Recall	82.26	81.56	87.04	83.62
*F*1	80.58	82.09	86.80	83.16

**Table 2:** Operating effect comparison.

Contrast indicators	Knowledge graph monitoring program	Relational database monitoring program
Accuracy of monitoring combined use of two drugs	95.1%	91.3%
Accuracy of monitoring combined use of three or more drugs	86.4%	68%
Accuracy of monitoring symptomatic administration	92%	No such function
Accuracy of repetitive efficacy medication monitoring	100%	100%
Accuracy rate of monitoring syndrome differentiation and treatment	83.1%	80%
Accuracy of dosage monitoring	97%	70%
Simultaneous monitoring of the number of electronic medical records	200	1
Conflict response time of two drugs prescribed online	0.8 S	1.3 S
Conflict response time of three or more drugs prescribed online	1.4 S	5.1 S
Background support for the number of Chinese patent medicines	Up to 20,000 species	8,000 species

## Data Availability

Datasets generated during the current study are available from the corresponding author upon reasonable request.

## Abbreviations

CPM: Chinese patent medicines
BERT: Bidirectional encoder representations from transformers
TCM: Traditional Chinese medicine.

## References

[1] World Health Organization (1993). How to investigate drug use in health facilities: selected drug use indicators. http://apps.who.int/iris/bitstream/10665/60519/1/WHO_DAP_93.1.pdf.

[2] Kesselheim A. S., Campbell E. G., Schneeweiss S. (2015). Methodological approaches to evaluate the impact of FDA drug safety communications. *Drug Safety*.

[3] Freifeld C. C., Brownstein J. S., Menone C. M. (2014). Digital drug safety surveillance: monitoring pharmaceutical products in twitter. *Drug Safety*.

[4] De Vries S. T., Van D. S. M. J. M., Van Der Sar M. J. M. (2018). Safety communication tools and healthcare professionals’ awareness of specific drug safety issues in Europe: a survey study. *Drug Safety*.

[5] Vries S. D., Van D., Cupelli A. (2017). Communication on safety of medicines in Europe: current practices and general practitioners’ awareness and preferences. *Drug Safety*.

[6] Haag J. D., Davis A. Z., Hoel R. W. (2016). Impact of pharmacist-provided medication therapy management on healthcare quality and utilization in recently discharged elderly patients. *American Health & Drug Benefits*.

[7] Khan N. U. Z., Rasheed S., Sharmin T. (2015). Experience of using mHealth to link village doctors with physicians: lessons from Chakaria, Bangladesh. *BMC Medical Informatics and Decision Making*.

[8] Jian W., Gao L. J. (2015). Influencing analysis of the national essential medicines policy on rational usage of medicines in primary health facilities. *Chinese Hospital Management*.

[9] Ma Z. (2019). Analysis of adverse drug reaction report of Chinese patent medicine from 2015 to 2017 in our hospital. *The Medical Forum*.

[10] Wang S. b. (2013). The development status and countermeasure of traditional Chinese medicine in Jiangxi province township hospitals. *Journal of Jiangxi University of Traditional Chinese Medicine*.

[11] Su Q. (2018). Analysis on monitoring of clinical irrational drug use by prescription automatic screening system. *Evaluation and Analysis of Drug-Use in Hospitals of China*.

[12] Wang H., Yang Z., Mao X. (2018). Construction of a rational drug use monitoring ecosystem based on regional medical big data. *Pharmaceutical Care and Research*.

[13] Lu C., Lin L., Gu Y. (2017). Drug monitoring system and data analysis for patients with renal impairment in a hospital. *Chinese Journal of Hospital Pharmacy*.

[14] Angraal S., Krumholz H. M., Schulz W. L. (2017). Blockchain technology:Applications in health care. *Circulation:Cardiovascular Quality and Qutcomes*.

[15] Dagher G. G., Mohler J., Milojkovic M., Marella P. B. (2018). Ancile: privacy-preserving framework for access control and interoperability of electronic health records using blockchain technology. *Sustainable Cities and Society*.

[16] Xue T., Fu Q., Wang C., Wang X. (2017). A medical data sharing model via blockchain. *Acta Automatica Sinica*.

[17] Chen D., Zhi L. (2015). Fog computing based hospital information service system. *Computer Science*.

[18] Wu X., Xie G., Cai C. (2018). Discussion on effects of PASS on monitoring the rationality of clinical medication in xiamen haicang hospital. *Evaluation and Analysis of Drug-Use in Hospitals of China*.

[19] Bao W., Tao Y., Wang L. (2018). Development,Problems and countermeasures of TCM international standardization. *World Chinese Medicine*.

[20] Wang C., Yang Y., Hu J., Lin M. (2018). Status, problems and relevant policy elucidation of standardization of traditional Chinese medicine. *China Journal of Traditional Chinese Medicine and Pharmacy*.

[21] Tang M., Liu T., Liu C. (2018). Design and implementation of health management system based on mobile application. *Computer Science and Application*.

[22] Hu X., Li L., Guo Q., Guo G. (2019). Analysis and discussion on the database model for medication consultation in a TCM hospital. *China Pharmacist*.

[23] Wang C., Gao M., He X. Challenges in Chinese knowledge graph construction.

[24] Yoshua B., Antoine B., Jason W. (2014). A semantic matching energy function for learning with multi-relational data application to word-sense disambiguation. *Machine Learning*.

[25] Chen D., Yin S., Le J. (2017). A link prediction model for clinical temporal knowledge graph. *Journal of Computer Research and Development*.

[26] Tong Y., Liu J. (2015). Research on the construction of big knowledge graph for traditional Chinese medicine. *China Digital Medicine*.

[27] Tong R., Sun C., Wang H. (2016). Construction of traditional Chinese medicine knowledge graph and its application. *Journal of Medical Intelligence*.

[28] Zhang D., Xie Y., Man L., Shi C. (2017). Construction of knowledge graph of traditional Chinese medicine based on the ontology. *Technology Intelligence Engineering*.

[29] Wang H., Peng S., Jin G., Chen D. (2014). Data visualization of traditional Chinese medicine based on random forest. *Journal of System Simulation*.

[30] Chinchor N. MUC-7 named entity task definition.

[31] Golshan P. N., Dashti H. R., Azizi S. (2018). A study of recent contributions on information extraction. https://arxiv.org/abs/1803.05667.

[32] Devlin J., Chang M. W., Lee K. (2018). Bert: Pretraining of deep bidirectional transformers for language understanding. https://arxiv.org/abs/1810.04805.

[33] Saffran J. R., Newport E. L., Aslin R. N. (1996). Word segmentation: the role of distributional cues. *Journal of Memory and Language*.

